# (Mis)perceiving cooperativeness

**DOI:** 10.1016/j.copsyc.2021.06.020

**Published:** 2022-02

**Authors:** Charlotte S.L. Rossetti, Christian Hilbe, Oliver P. Hauser

**Affiliations:** a Max Planck Institute for Evolutionary Biology, Plön, Germany; b Department of Economics, University of Exeter, Exeter, EX4 4PU, UK

**Keywords:** Perception, Cooperation, Social behavior, Cues

## Abstract

Cooperation is crucial for the success of social interactions. Given its importance, humans should readily be able to use available cues to predict how likely others are to cooperate. Here, we review the empirical literature on how accurate such predictions are. To this end, we distinguish between three classes of cues: behavioral (including past decisions), personal (including gender, attractiveness, and group membership) and situational (including the benefits to cooperation and the ability to communicate with each other). We discuss (i) how each cue correlates with future cooperative decisions and (ii) whether people correctly anticipate each cue's predictive value. We find that people are fairly accurate in interpreting behavioral and situational cues. However, they often misperceive the value of personal cues.

When people cooperate, they make an individual effort to benefit others [1]. Such voluntary acts of costly cooperation are crucial for most social interactions, affecting the well-being of families, the success of scientific collaborations, and the output of team work. Cooperative behavior can be explored from various angles. Theoretical studies analyze which social environments are most conducive to cooperation [2, 3, 4]. Similarly, experimental work explores under which conditions people actually cooperate and how they adapt their own behavior to the actual or expected behavior of their peers [5, 6, 7, 8]. Here, we review this literature, focusing on the following two questions: how do people use available cues to predict the cooperativeness of their interaction partners ahead of an interaction, and which cues are most reliable for predicting cooperativeness?

We distinguish between three broad categories of cues that may be used to make such predictions. (i) *Behavioral cues* refer to the interaction partners' past actions. Examples of such cues include whether these partners cooperated on previous occasions or whether they enforced cooperative social norms. (ii) *Personal attributes* comprise, for example, the interaction partner's gender or perceived attractiveness, among other characteristics. (iii) *Situational cues* define the environment in which the individual's next interaction takes place, including how costly cooperation will be and whether pre-play communication is possible. For each of the three categories, we ask whether the given cue is in fact a reliable predictor of cooperativeness and whether people judge the cue's predictive value accurately. Although the existing literature covers a wide range of relevant cues, there also exist notable gaps in the literature, which we encourage scholars to explore further.

There are various ways to elicit how individuals perceive each other's cooperativeness. For the purpose of this review, we consider evidence from three approaches. The first approach is to elicit perceived cooperativeness directly by asking participants to estimate how likely others will cooperate. The two other approaches are more indirect by either asking participants to choose a group member for future interactions (i.e. partner selection) or by asking participants how much money they would transfer to the respective group member in a trust game [9]. Our assumption is that the more inclined a participant is to choose an interaction partner or to transfer more money to a partner, with whom they will interact in the future, the more cooperative they perceive the partner to be —and indeed, some empirical work has found this to be the case [10].

## Behavioral cues of cooperativeness

Perhaps the most immediate cue to predict future cooperative behavior is whether, and how often, the respective individual cooperated in the past. Experimental research suggests that people exhibit a stable and consistent ‘cooperative phenotype’: an individual's decision in one cooperative game is indicative of what that individual will subsequently do in different games [11,12]. Participants in laboratory experiments, in turn, seem to expect others to be consistent in their cooperative behavior. When people need to choose an interaction partner, they strongly prefer partners who have been cooperative in the past [13]. Similar evidence comes from studies on charitable giving and pro-environment behavior. Donors to charity are trusted more as well as chosen more often as interaction partners, and in many cases, they indeed turn out to be more cooperative in subsequent social dilemmas [14,15]; but see also [16].

Another — more indirect — cue of cooperative behavior is whether individuals previously engaged in the enforcement of social norms. As per this account, people who punish selfishness may signal that they are not selfish. To test this hypothesis, Jordan et al. [17] consider an interaction that consists of two stages. In the first stage, a ‘signaler’ witnesses a transgressor who refuses to help a recipient. The signaler can then decide whether to engage in third-party punishment by reducing the payoff of the transgressor. In the second stage, a ‘chooser’ decides how much money to send to the signaler in a trust game. The experiment shows that signalers who punish transgressors are indeed entrusted with more money, which turns out to be justified: these signalers also return more money to the chooser. Interestingly, when in the first stage signalers have a choice between punishing the transgressor or helping the recipient, signalers are less likely to punish. Instead, helping turns out to be the more frequently chosen (and more accurate) signal of trustworthiness. This result is in line with an earlier experiment reported by Rockenbach and Milinski [18]: when individuals need to choose group members for a cooperative task, they place more weight on how often group members cooperated, rather than how often they enforced cooperation. Overall, punishment may not necessarily be taken as a cue of altruism, as it may also imply aggressiveness. As a result, punishment is judged more appropriate if it is implemented by the entire group, rather than by a single individual [19].

Finally, another potential cue may come from how a person makes cooperative decisions. For example, based on a game-theoretic model, Hoffman et al. [20] suggest that people who collect additional information to carefully compare the advantages and disadvantages of a cooperative decision are considered less reliable and less cooperative. As per this account, people who deliberately refuse to learn payoff-relevant information may seem more committed to cooperation even when defection happens to be profitable. In line with this view, Jordan et al. [21] show that study participants who ignore the precise costs of cooperation are indeed (and accurately) predicted to be more trustworthy. Participants in turn seemed to be well-aware of the reputational benefit of strategic ignorance: when the cooperation costs can be learnt secretly, participants were more likely to do so. Similar evidence comes from a study reported by Levine et al. [22] who compare the reputational consequences of emotion-based versus reason-based decision-making. Players who state having made a decision based on emotion are perceived as more cooperative by their partner and indeed turn out to be more cooperative. Interestingly, however, players who state having used reason were not perceived as any less cooperative than a control group.

## Personal cues of cooperativeness

A second category of cues pertains to personal characteristics of individuals. The idea that visible characteristics are used as signals of cooperativeness has its roots in evolutionary biology: computer simulations and game-theoretic models suggest that individuals can use visible cues to identify potential cooperation partners. This theoretical work suggests that partner choice based on visible cues can in turn be an important mechanism for the evolution of cooperation [23].

Arguably, one of the most salient personal cues is gender. Although some scholars document gender differences in cooperativeness in the dictator game [24] and the prisoner's dilemma [25], the literature remains notoriously mixed [26, 27, 28]. Indeed, Exley et al. [29] show that gender only inconsistently predicts cooperativeness across seven economic games. Nevertheless, across all economic games, they find robust evidence that people consistently believe that women are expected to be fairer, more generous, and more cooperative than men, in line with studies reported by Aguiar et al. [30] and Brañas-Garza et al.[31].

Other cues about a person include their physical appearance, such as an interaction partner's face and facial expressions [32, 33, 34∗], their voice [35] and, in particular, their attractiveness [36,37]. Although there is no evidence that attractiveness is a reliable predictor of cooperativeness, good looks nonetheless positively impact people's perception of others in many domains of economic life, known as the ‘beauty premium’ [38]. Indeed, Anderoni and Petrie [36] and Wilson and Eckel [37] find that people expect more cooperation and reciprocation from attractive partners. Interestingly, when such expectations are not met, attractive interaction partners incur a ‘beauty penalty,’ receiving less reciprocation than less attractive players.

Another factor that may affect a partner's cooperativeness is wealth. Here, the experimental evidence remains inconclusive. For example, Piff et al. [39] find that subjects who consider their own socioeconomic rank to be low tend to be more generous and charitable. In contrast, Smeets et al. [40] report that millionaires are considerably more generous in dictator games than usual participants, especially if they are paired with a low-income partner. A similarly conflicting picture emerges on the level of perceived cooperativeness. Some experiments find that wealthy participants are perceived to be more trustworthy and cooperative [41,42], while at the same time, people seem to systematically underestimate the generosity of the extremely rich [43].

A person's political, religious, or ethical convictions can also serve as potential cues of cooperativeness. Research on political affiliation finds that left-leaning participants tend to cooperate more than right-leaning participants. However, the effect is small at best [44,45], and it seems to be moderated by the fact that left-leaning participants expect more cooperation from others [46]. Political ideology in turn shapes how people are perceived. Balliet et al. [47] find that among United States participants, Democrats are perceived as more cooperative by both sides of the political spectrum, even though this belief is inaccurate. Similar to political ideology, religiosity appears to be correlated with prosocial behavior [48,49]. As a result, when Christians show overt religious cues (e.g. a necklace with a cross), they are perceived as more trustworthy [50]. Finally, people are considered more trustworthy when they make deontological rather than consequentialist judgments [51,52]. Interestingly, however, deontological participants are not necessarily more cooperative [53].

More often than not, when it comes to social attributes, perceptions of cooperativeness are partially shaped by in-group bias, as group membership is itself an important cue. A large literature demonstrates that participants cooperate more with, and preferentially reward, ‘in-group’ members, both in the laboratory [54] and in the field [55]. Democrats and Republicans both tend to cooperate more with in-group members [47], and participants cooperate more with a partner that shares their nationality [56]. However, group membership may largely serve as a coordination device: although participants do not believe that in-group members are intrinsically more cooperative than others, Balliet et al [57] find that more cooperation is expected from in-group members.

## Situational cues of cooperativeness

Our final category of cues covers situational aspects surrounding a cooperative decision. These include factors outside an individual's control that are often determined by the structure or context of the interaction. For example, some social interactions create more mutual benefits to the participants than others. In laboratory studies, these factors can be studied in isolation, holding everything else constant. For instance, in the repeated prisoner's dilemma, people tend to be more cooperative when the mutual benefit of cooperation increases [58]. This is consistent with evidence from a study reported by Charness et al. [59]: as the mutual benefit of cooperation increases, participants also expect to see more cooperativeness, especially those participants who later choose to cooperate. This suggests that individuals are able to ‘read’ a situation and that they adjust their willingness to cooperate accordingly.

There has been a debate on whether cooperativeness is affected by whether or not decisions need to be made under time pressure. One account holds that when individuals are forced to decide quickly, they tend to be more cooperative [60]. However, the causal evidence is mixed [61]. Alternatively, it has been argued that fast decisions may not necessarily result in more cooperation. Instead, it may result in more extreme outcomes, either toward cooperation or defection [62]. This is also what participants themselves seem to expect: when asked to predict the outcome of a fast cooperation decision, participants are more likely to expect an extreme (but not necessarily a cooperative) outcome [63].

Finally, communication has long been found to enhance cooperation [64]. However, there is debate as to what is the precise mechanism that allows communication to be favorable. He et al. [65] rule out several potential explanations, namely, that communication reduces social distance or that it offers opportunities to make promises. Instead, they argue that most importantly, communication allows people to recognize the other person as a cooperative type. Indeed, Sparks et al [66] find that people can accurately predict cooperative behavior in a prisoner's dilemma after only a short in-person interaction, even when participants do not discuss the game itself.

## Conclusion and future directions

People are quick to form an impression, sometimes in just a few milliseconds [32], but these impressions do not need to be reliable. In this review, we have summarized how individuals perceive different cues to predict others’ cooperativeness. Among the three categories of cues we considered, people most accurately use behavioral and situational cues to predict future cooperative behavior. In contrast, predictions seem to be least accurate when they are based on personal attributes. In fact, with the possible exception of political affiliation and religiosity, personal attributes are often not a good predictor of actual cooperation behavior, yet many such attributes — such as gender and attractiveness — are nonetheless (and inaccurately) perceived as predictors of cooperativeness.

These findings raise a number of interesting questions. For example, theoretical work could explore which kinds of environments and cues allow people to form reliable expectations. Similarly, experimental work could investigate how persistent certain misperceptions are and whether they disappear with more experience. Although we summarized results on a number of different cues, several others seem to have received limited attention, including age and ethnicity.

Further interesting problems arise when several (possibly conflicting) cues are available or when people need to aggregate cues from different domains. For example, Kumar et al. [56] show that when people learn both their interaction partner's nationality and gender, the former cue becomes dominant: people cooperate more with own nationality partners, and they expect own nationality partners to be more cooperative.

More generally, any given cue seems to become less relevant once more salient information — such as actual cooperative behavior — is available [17,36,67,68]. These findings suggest that people rank different cues according to each cue's predictive value. They only make use of unreliable cues when no other cues are available.

## Conflict of interest statement

Nothing declared.
